# In-House Manufacturing of a Translucent Three-Dimensional Model and Surgical Guide for Marginal Mandibulectomy

**DOI:** 10.7759/cureus.54771

**Published:** 2024-02-23

**Authors:** Yutaro Kondo, Masashi Kimura, Mizuki Hyodo, Kengo Hashimoto, Mitsuo Goto

**Affiliations:** 1 Department of Oral and Maxillofacial Surgery, Ogaki Municipal Hospital, Ogaki, JPN; 2 Department of Maxillofacial Surgery, School of Dentistry, Aichi Gakuin University, Nagoya, JPN; 3 Department of Oral and Maxillofacial Surgery, Toyokawa City Hospital, Toyokawa, JPN

**Keywords:** 3d printing, head and neck cancer surgery, surgical guide, mandibulectomy, computer-assisted surgery

## Abstract

In recent years, intraoperative surgical guides have been widely used in oral and maxillofacial surgery to navigate the resection sites. However, most of them are designed for segmental mandibulectomy and determine only the anterior-posterior cutting sites. In the case of marginal mandibulectomy, the depth and angle of the resection need to be considered in addition to the anterior-posterior cutting site. This report describes a method for creating a translucent mandible model with a colored tumor that enables visualization of the tumor depth and a surgical guide for marginal mandibulectomy with a planned resection angle. If accurate surgical planning and intraoperative navigation are established using this method, personalized surgery is realized according to tumor features and hence avoids over- or under-resection.

## Introduction

Recently, virtual surgical planning (VSP) has been widely used in oral and maxillofacial surgery [1-3]. With these advancements, computer-assisted design (CAD) and computer-aided manufacturing techniques have now become essential for oral and maxillofacial surgeons [2,4]. In addition, the usefulness of in-house manufacturing, in which three-dimensional (3D) printing is performed within its own facility, has been demonstrated in recent years [5-7].

Intraoperative surgical guides for tumor resection have been widely used to define the region of a planned resection [2,5,6]. However, most of them are designed for segmental mandibulectomy and determine only the anterior-posterior cutting sites [5,6]. In the case of marginal mandibulectomy, the depth and angle of the resection need to be considered in addition to the anterior-posterior cutting site. However, it is impossible to confirm the accurate depth of bone invasion intraoperatively, which may lead to over- or under-resection.

Therefore, we developed a translucent mandible model with a colored tumor that enables visualization of the tumor depth within the model. Moreover, using this model, VSP was performed creating a surgical guide with an optimal resection angle for marginal mandibulectomy, which is reported here.

## Case presentation

**Figure 1:**
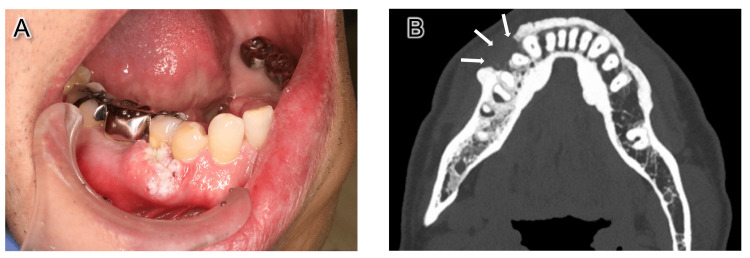
Intraoral photograph and CT image. (A) Clinical examination showed a mass measuring 20 × 15 mm in the right lower premolar region. (B) CT image of the tumor area. Bone invasion was seen (white arrows).

A 62-year-old male patient presented to our hospital with a chief complaint of an ulcer on the right lower gums. Clinical examination showed a mass measuring 20 × 15 mm in the right lower premolar region, as well as palpable enlargement of the right neck lymph nodes (Figure 1A). Computed tomography (CT) examination revealed bone resorption of the tumor region (Figure 1B) and two enlarged submandibular lymph nodes. An incisional biopsy was performed, and a pathological diagnosis indicated a well-differentiated squamous cell carcinoma (SCC). Therefore, a right marginal mandibulectomy and neck dissection were planned, with a diagnosis of right mandibular gingival SCC (cT2N2bM0). The surgical margin was planned to be set at 12 mm from the tumor, slightly wider than the typical safety margin of 10 mm considering the rapid growth of the tumor and the results of preoperative assessments based on our previous study [8].

**Figure 2:**
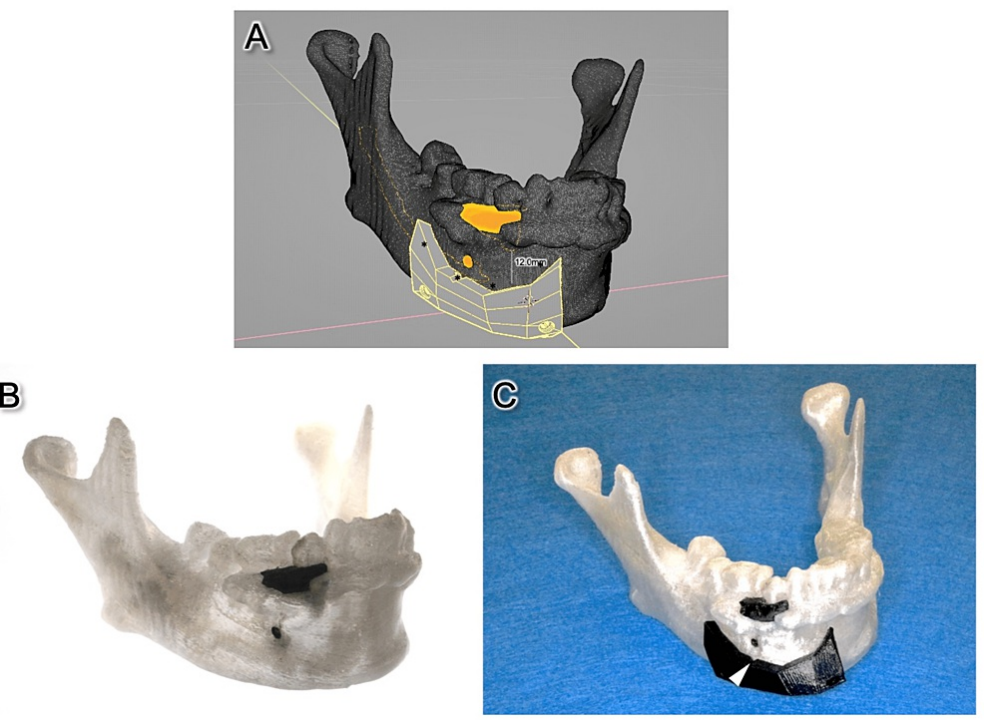
Virtual surgical planning and created translucent 3D model and surgical guide. (A) Virtual surgical planning and design of the surgical guide. The upper surface of the created surgical guide not only defines the depth of the resection site but also indicates the insertion angle of the bone saw according to the 3D depth of the tumor. (B) Created translucent 3D model showing the tumor location. (C) Created surgical guide by VSP. A notch below the mental foramen was used as a marker for positioning the surgical guide (arrowhead).

To perform accurate resection, we developed a translucent mandible model with a colored tumor to visualize the tumor depth and a surgical guide with a defined resection angle. First, the patient’s contrast-enhanced CT images with a slice thickness of 1.0 mm were transferred to a disk as digital imaging communication in medicine (DICOM) data. Subsequently, the data were exported to 3D Slicer (Boston, USA), an open-source software, and semiautomatic segmentation of the mandible was performed. Then, the tumor region was carefully selected by manual segmentation. After segmentation was completed, these two segments were separately exported as stereolithography (STL) files and imported into the free CAD software, Blender (Blender Foundation, Amsterdam, The Netherlands), and the 3D tumor location was carefully referred, and the surgical guide was created with a 12-mm safety margin. The upper surface of the created surgical guide not only defines the depth of the resection site but also indicates the insertion angle of the bone saw according to the 3D depth of the tumor (Figure 2A). Subsequently, four screw holes were created to secure the surgical guide to the mandible intraoperatively. The created mandible model and surgical guide were saved as an STL file and printed on a 3D printer, Flashforge Creator Pro 2 (Flashforge, CA, USA) (Figures 2B-2C). The mandible model and surgical guide were sterilized with ethylene oxide gas.

**Figure 3:**
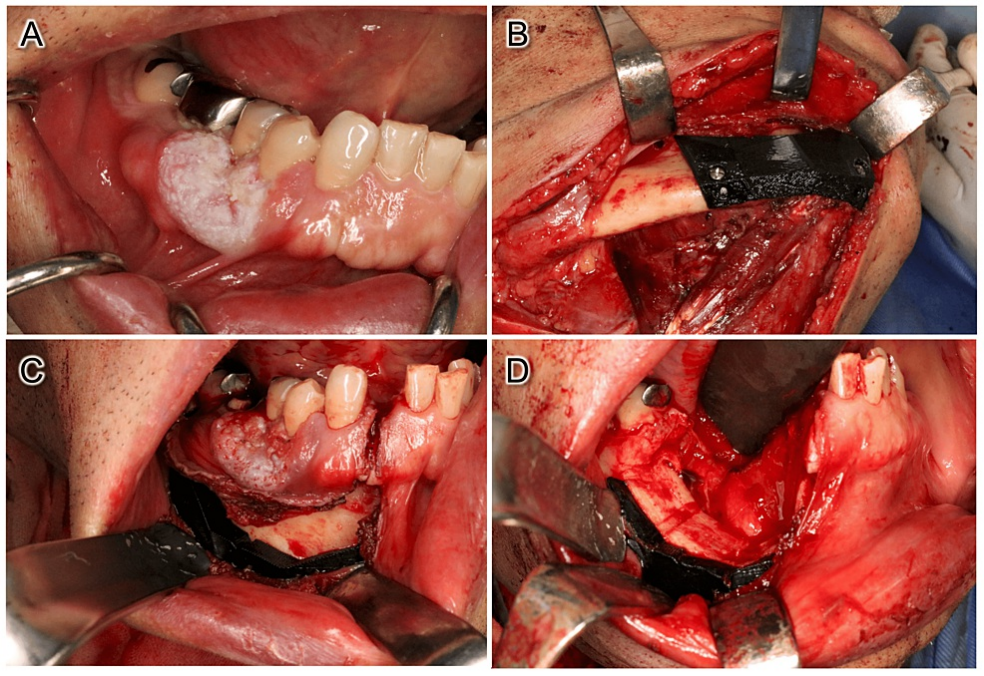
Intraoperative photographs. (A) Intraoral image at the time of surgery. The tumor showed a tendency to increase in size since the initial examination. (B) Surgical guide was fixed with two screws. (C) Intraoral image after fixation of the surgical guide. (D) Intraoral image after resection.

**Figure 4:**
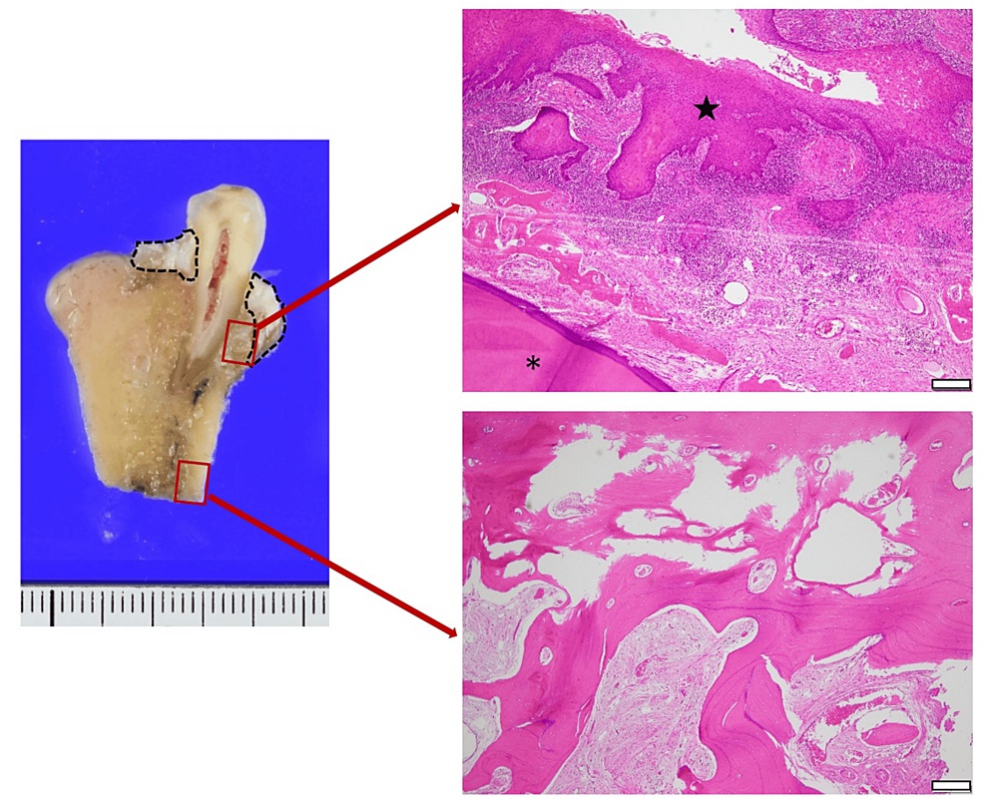
Cross-sections of the resected tissue and H&E-stained images. Cross-sections of the resected tissue (oral mucosa was stripped) and H&E-stained image of the bone invasion area and resection margin (*: tooth, ★: tumor, scale bar = 50 𝜇m). The dotted line indicates the area of bone invasion, and histopathological examination of the resection margins showed no tumor cells, suggesting that the resection was performed with an adequate margin of safety.

In surgery, neck dissection was first performed, followed by marginal mandibulectomy. Briefly, a resection line was made with a 12 mm safety margin in the oral cavity. Then, the periosteum of the inferior border of the mandible was incised and contiguous with the intraoral surgical field, and the surgical guide was fixed with screws (Figures 3A-C). At that time, the mental nerve was identified, and a notch of the surgical guide created as a marker was used to position the surgical guide. Then, transoral marginal mandibulectomy was performed using an oscillating saw following the inclination angle of the surgical guide as planned (Figure 3D). Histopathological examination of the resected tissue revealed no evidence of tumor cells in the resected margins. Furthermore, cross-sections of the resected tissue indicated that the resection was performed consistent with the preoperative plan (Figure 4).

## Discussion

In the present method, the mandible and tumor were exported as two STL files, and the translucent 3D model and surgical guide were created to enable VSP while accurately determining tumor depth. Furthermore, because the exact tumor depth can be determined three-dimensionally, it is possible to define the resection angle in the surgical guide according to the tumor depth. Intraoperative surgical guides for tumor resection have been widely used to define the planned resection site [2,5,6]. However, most of them are designed for segmental mandibulectomy and determine only the anterior-posterior cutting sites [5,6]. On the other hand, Dong et al. have reported a surgical guide for marginal mandibulectomy [9]. Although they used a surgical guide for a patient with recurrent ameloblastoma of the mandibular body, the exact resection angle according to the tumor depth was not specified. Thus, to the best of our knowledge, the present study is the first to report on the use of a surgical guide for marginal mandibulectomy to define the resection angle. We also performed marginal mandibulectomy using an oscillating saw, and it was thought to contribute to accurate and easy cutting along the angle of the surgical guide. This technique also may be a useful method for using the present surgical guide. Furthermore, although the conventional single-color 3D model provides limited information, the translucent 3D model can provide additional information, such as the relationship between the extent of bone invasion and the surgical guide. This preoperative examination allows additional modifications, such as adjusting the cutting angle and size of the guide to the 3D-printed surgical guide.

In addition, the cost of outsourcing a mandibular model similar to the one described here is reported to exceed $1,000, although, on a case-by-case basis, fees for VSP services are also reported to start at about $1,000 [10]. Contrarily, the equipment used in this report was extremely inexpensive, costing about $700 to install and only $10 for materials to print the translucent 3D model. Furthermore, the time required for discussion, planning, and transportation to create the commercial model may range from a few days to four weeks [9,10]. This drawback is unacceptable for patients with malignant tumors. The turnaround time for in-house production was approximately one to two days, including software processing and 3D printing.

This surgical procedure has several limitations. First, this is a single-case report, and the accuracy of the VSP needs to be verified by accumulating more cases. Second, the processes to create the 3D model are somewhat complex, and a certain amount of time may be needed to be able to proficiently use the software. Furthermore, differences in the accuracy of VSP due to the proficiencies of designers and the performance of the 3D printers should be considered. Therefore, we believe that a large-scale study is warranted to evaluate the accuracy of VSP.

## Conclusions

If accurate surgical planning and intraoperative navigation are established using this surgical guide, personalized surgical planning will be feasible, demonstrating an accurate safety margin in the tumor depth to avoid over- or under-resection.
